# Focusing on Global Environmental Health

**DOI:** 10.1289/ehp.114-1665393

**Published:** 2006-11

**Authors:** David A. Schwartz, William J. Martin

**Affiliations:** Director, NIEHS and NTP, E-mail: david.schwartz@niehs.nih.gov; Director, Office of Translational Research, E-mail: wjmartin@niehs.nih.gov

From the problems of arsenic in drinking water, to indoor air pollution from burning of biomass as fuel, to lead’s effects in children, global environmental health problems are enormous and daunting. Some of these same or equally menacing hazards threaten the health of many in the United States, particularly and often disproportionately the poor; however, the scale of such threats here pales in comparison to that of much of the world. One irony of this situation is that the places where the environmental health crises seem most dire may offer the best opportunities for research toward understanding the problem and identifying solutions that ultimately improve world health. And we know, too, that when it comes to many environmental health problems, no one nation can stand in isolation; the action or inaction of those in one place has the potential to affect the lives of those far distant. We are currently engaged in a process of revitalizing and refocusing the NIEHS’s efforts in global environmental health to maximize our potential to prevent disease and improve human health.

The NIEHS has a long history of partnering with universities and research institutes to support investigations into environmental health problems wherever they occur in the world. These partnerships have led to a better understanding of both the extent and mechanisms of problems such as arsenic in drinking water in Bangladesh, the link between aflatoxin and liver cancer in China, the effect of lead on children’s neurodevelopment in Mexico, and many others. The NIEHS is committed to its ongoing partnerships with the Fogarty International Center and other NIH institutes and centers as global issues in environmental health continue to emerge that potentially impact the American public.

However, the successful development of new initiatives in global environmental health will require new partnerships, as well, partnerships that are equitable to all parties and based in mutual understanding, experiences, and commitment to addressing the scientific questions at hand. As a first step, we have started a new program that will offer funding to allow junior-level faculty scientists from developing nations to conduct research in NIEHS-funded laboratories. We are also exploring ways to support U.S.-based investigators to work in the laboratories of foreign scientists.

The solutions to fundamental problems in environmental health require a global effort to better understand the causes and impact of these hazards on human health. The prevention of these diseases requires that we learn how to control exposures and identify those at risk of developing disease, as well as those in the early, reversible stage of illness. These advances will be facilitated in a collaborative research environment. Armed with this understanding, we believe the best approach for our program in global environmental health will be to focus our efforts on identifying a major problem in human health that is caused by a compelling environmental exposure—and thus is ultimately correctable—and working to address that problem through global partnerships.

For example, childhood respiratory infections represent a preventable disease that causes substantial morbidity and mortality and is overrepresented in developing countries. The indoor burning of biomass fuels combined with outdoor air pollution very likely contributes to the deaths of children throughout the world each year from preventable respiratory infections and progressive airway disease. And although excess cardiovascular and pulmonary deaths have been linked to exposure to air pollution in many developed countries, we still don’t understand the fundamental pathogenesis of these diseases or why some individuals are more susceptible to these complications. Focusing on understanding childhood respiratory infections might create an opportunity to substantially enhance our understanding of all of these diseases and lead to measurable gains in human health in both the developing and developed world. This same scenario could likely prove true for many other environmental health problems as well, any of which may represent opportunities for the NIEHS to understand human biology and improve human health both here and abroad.

In early 2007, we will be hosting a workshop to focus intensively on identifying global environmental health problems that may give us the greatest chance of better understanding human biology and improving human health in the not-too-distant future. We’re asking leaders in the environmental health sciences and other disciplines from around the world to help us pursue this question, as well as address the potential size and scope of the effort, the barriers to success, the methodologies that should be followed, and the possible partnerships that would facilitate an NIEHS-led effort. As global leaders in the environmental health sciences, we are obliged to understand how the environment contributes to inequities in world health and find lasting solutions to these problems.

## Figures and Tables

**Figure:**
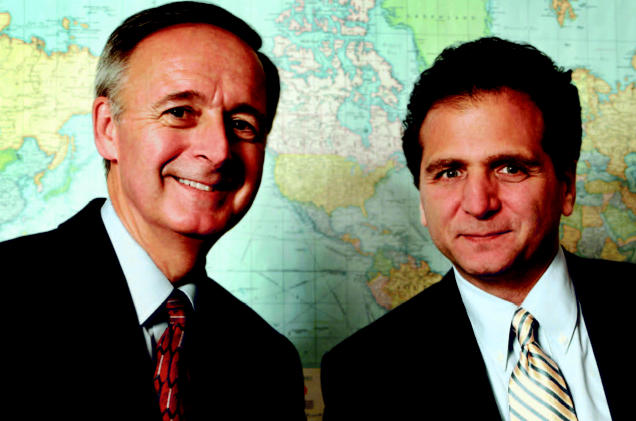
(left to right) Bill Martin and David Schwartz

